# Characterization of the Integration and Modular Excision of the Integrative Conjugative Element PAISt in *Streptomyces turgidiscabies* Car8

**DOI:** 10.1371/journal.pone.0099345

**Published:** 2014-06-13

**Authors:** Jose C. Huguet-Tapia, Dawn R. D. Bignell, Rosemary Loria

**Affiliations:** 1 Department of Plant Pathology, University of Florida, Gainesville, Florida, United States of America; 2 Department of Biology, Memorial University of Newfoundland, St. John's, Newfoundland and Labrador, Canada; Virginia Tech, United States of America

## Abstract

PAISt is a large genomic island located in the chromosome of the plant pathogen *Streptomyces turgidiscabies* Car8. The island carries clustered virulence genes, transfers to other *Streptomyces* species, and integrates by site-specific recombination at the 8 bp palindrome TTCATGAA. The palindrome is located at the 3′ end of the bacitracin resistance gene (*bacA*). We demonstrate that PAISt is able to excise in modules by recombination of one internal and two flanking palindromic direct repeats. The gene *intSt* located at the 3( end of PAISt encodes a tyrosine recombinase. Site-specific recombination activity of *intSt* was tested and confirmed by heterologous expression in *Streptomyces coelicolor*. Comparative analysis of PAISt homologues in *Streptomyces scabies* 87–22 and *Streptomyces acidiscabies* 84–104 indicates that these islands have been fixed by sequence erosion of *intSt* and the recombination sites.

## Introduction

Integrative conjugative elements (ICEs) are mobile genetic elements found in prokaryotes that play an important role in lateral gene transfer (LGT) [1], [2]. These genetic elements are mainly circular structures without self-replication properties [1]. ICEs move by conjugation from a donor to a recipient and integrate in specific locations in the chromosome [1], [2]. The process of integration is conducted by a site-specific recombinase, InT [1],encoded within the ICE, and occurs at the bacterial attachment site (*attB*) in the chromosome through recombination of this site with the ICE attachment site (*attP*) [1].

Once integrated in the host chromosome, two new recombination sites, *attL* and *attR*, flank the ICE. These *att* sites result from conservative recombination of *attB* and *attP*. This process leaves the recombination-core-sequence intact. Therefore, the integrase can excise the ICE by recombining with the flanking *att* sites again. The excision reconstitutes the circular structure of the ICE. In this state the element can integrate again in the chromosome or it can be transferred to a new host by conjugation, using a set of DNA mobilization proteins encoded within the ICE [1]. Most ICEs transfer as a single DNA strand; however, several conjugative elements in *Streptomyces* and related actinobacteria transfer as double stranded DNA [3], [4].

Several species of pathogenic *Streptomyces* infect underground plant structures such as roots and tubers. Most notably, pathogenic *Streptomyces* species cause the economically significant disease potato scab [5]. It is believed that LGT plays an important role in the evolution of plant pathogenic streptomycetes [6], [7]. The best-characterized instance of LGT in these pathogens is the large pathogenicity island, PAISt, which exists in *S. turgidiscabies* [8], [9], a pathogenic species reported for the first time on the island of Hokkaido, Japan in the late 1900s [6].

**Figure 1:**
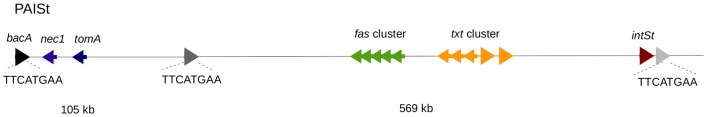
Schematic representation of the PAISt in *S. turgidiscabies* Car8. Copies of the 3′ end of the bacitracin resistance gene (*bacA*) delimit the element in two modules of 105 Kb and 569 Kb. The virulence genes *nec1* and *tomA* are located in the first module and the fasciation (*fas*) and thaxtomin (*txt*) biosynthetic clusters are located in the second module. The putative integrase (*intSt*) is located at the 3′ end of the island. The 8 bp palidromic repeats are shown within the *bacA* gene and its truncated copies.

Previous studies have revealed that PAISt is a large genomic island of 674 Kb with features typical of ICEs [8], [9]. The genomic island is flanked by two 8 bp palindromes (TTCATGAA). One of them (*attL*) is located at the 3′ end of the *bacA* gene and the other (*attR*) is located within a 141 bp duplication of the 3′ end of *bacA* (Figure 1). PAISt also contains an internal 8 bp palindrome located within a third copy of the 3′ end of the *bacA (attI)*. The distribution of the *att* sites in PAISt divides the element into two different size modules (Figure 1). The first module of 105 Kb encodes a tomatinase (*tomA*) and a necrogenic protein, *nec1*; both are secreted proteins implicated in virulence [10], [11]. The second module of 569 Kb contains the biosynthetic pathway for the phytotoxin thaxtomin (*txt*) [12], a plant fasciation biosynthetic pathway (*fas*) [13], and a putative tyrosine recombinase (*intSt*) [8].

The PAISt can mobilize from *S. turgidiscabies* and integrate into the chromosome of *S. coelicolor*, *S. diastochromogenes* and *S. lividans* during mating [9]. In some instances, such integration transfers the pathogenic phenotype. The integration of PAISt into the new hosts occurs at the eight-base palindromic sequence TTCATGAA located at the 3′ end of the bacitracin resistance gene (*bacA*) [9]. Streptomycete transconjugants contain the complete 674 Kb sequence of the PAISt or the 105 Kb module, integrated in the recipient's chromosome.

The distribution of the *att* sites within the PAISt, the presence of *intSt* at the 3′ end of the element, and the evidence of partial transfer of the island to new hosts suggest that the PAISt may excise as modules. The process of excision may follow a site-specific recombination at the *att* sites driven by *intSt*. In this study, we show evidence of the modular excision of PAISt and we characterize *intSt*. We also suggest a model for the evolution of PAISt and its homologs in other plant pathogenic *Streptomyces* species.

## Materials and Methods

### Bacterial Strains and Culture Conditions

*Escherichia coli* strains (Table 1) were cultured in Luria broth (LB) and/or Luria agar (LA) media at 37°C. *Streptomyces* strains (Table 1) were cultured at 28°C using International *Streptomyces* Project 4 (ISP4) agar medium, mannitol-soya flour (MS) agar and tryptic soy broth (TSB). Concentrations of antibiotics used in the growth media are as follows: for *E. coli*, chloramphenicol (25 µg/ml), kanamycin (50 µg/ml), hygromycin B (100 µg/ml), and apramycin (100 µg/ml); for *Streptomyces* strains, chloramphenicol (25 µg/ml), kanamycin (25 µg/ml) and nalidixic acid (25 µg/ml).

**Table 1:** Bacterial strains and plasmids used in this study.

Strains	Genotype or comments	Source or reference
*E. coli* strains		
*E. coli* ET12567	Non-methylating host for transfer of DNA into *Streptomyces* spp. *dam* ^−^, *dcm* ^−^ *cam^R^*	[32]
*E. coli* DH5α	F- general cloning host	Gibco-BRL, Grand Island, NY, USA
*Streptomyces* strains		
*S. coelicolor* A3(2)	Wild type	
*S. coelicolor* Δ*bacA*	*S. coelicolor* A3(2) with *bacA* deletion. *kan* ^R^	This study
*S. turgidiscabies* Car8	Wild type	[6]
*S. scabies* 87–22	Wild type	[33]
*S. acidiscabies* 84–104	Wild type	[34]
**Plasmids**		
pIJ10257	Plasmid backbone for construction of derivatives. *hyg* ^R^	[35]
pIJamp001	Derivate of pIJ10257. *amp* ^R^, *hyg* ^R^	This study
pIJintSt	pIJamp001 derivate containing the coding sequence of *intSt* under control of the constitutive promoter *ermE*p* plus the *attR*. *amp* ^R^, *hyg* ^R^	This study
pIJintStatt(−)	pIJintSt derivate without the *attR* site. *amp* ^R^, *hyg* ^R^	This study
pIJintStY407F	pIJintSt derivate with a point mutation in *intSt* (Y407F). *amp* ^R^, *hyg* ^R^	This study
pIJintStR375K	pIJintSt derivate with a point mutation in *intSt* (R375K). *amp* ^R^, *hyg* ^R^	This Study
pIJintStR209K	pIJintSt derivate with a point mutation in *intSt* (R209K). *amp* ^R^, *hyg* ^R^	This Study
pIJattR+	pIJamp001 with *attR*. *amp* ^R^, *hyg* ^R^	This study

DNA methylation: Mutants defective in adenine methyltransferase *dam* ^−^. Mutants defective in cytosine methyltransferase *dcm* ^−^. Antibiotic resistance: chloramphenicol *cam^R^*, ampicillin *amp* ^R^ and hygromycin *hyg* ^R^.

### Plasmid Construction

Plasmid pIJamp001 is a derivative of the 6,108 bp plasmid pIJ10257 (Table 1), in which the phage integrase φBT1 and its integration site were replaced by the ampicillin resistance gene (*amp*) using the NcoI and EcoRV restriction sites. This replacement generated a plasmid of 4,768 bp. To construct pIJintSt (Table 1), the 1,401 bp coding sequence of *intSt* and the 41 bp downstream regions were amplified from *S. turgidiscabies* Car8 by PCR using primers intF and intR (Table 2). The 8 bp palindrome TTCATGAA is downstream of *intSt* and is the site of recombination for PAISt. Primers intF and intR (Table 2) contain the NdeI and HindIII restriction sites. Consequently, the PCR product was cloned into the NdeI-HindIII sites in plasmid pIJamp001 and positioned under control of the strong constitutive promoter *ermE*p* [14]. This process generated a plasmid of 6,230 bp containing *intSt* that is constitutively expressed. Plasmid pIJintStatt(−) (Table 1) is also a derivative of pIJamp001 but only contains the open reading frame of *intSt* amplified with primers intF and intX (Table 2).

**Table 2:** List of primers used in this study.

Primer	Nucleotide sequence	Purpose
intF	GGTCGA**CATAT** *G*CCCTACATCGAGTGGC	Amplification of *intSt* plus *attR* in *S. turgidiscabies* Car8.
intR	AGTG**AAGCTT**CGGCATGAACGACTTGGTCG	
intX	AGTG**AAGCTT**CTATGACCACTTAGCCTTG	Amplification of *intSt* in *S. turgidiscabies* Car8
FintStYF	GAGGGGTTG**TTC**TCCAACGTGACA	Point mutation for Y407F residue in *intSt*
RintStYF	TGTCACGTTGGA**GAA**CAACCCCTC	
FintStRK1	TACCTGATG**AAG**CACGGACAC	Point mutation of R209K residue in *intSt*
RintStRK1	GTGTCCGTGC**TTC**ATCAGGTA	
FintStRK2	ACCGGCATG**AAG**CCCGCTGAG	Point mutation of R375K residue in *intSt*
RintStRK2	CTCAGCGGGC**TT**CATGCCGGT	
a	CTCACCAACAAGGCGATGCG	Detection of circular structures in *S. turgidiscabies* Car8
b	TGACCCGCTCTACCCTCTGT	
c	GGGACGGTCTGATCTACGGC	
d	TGCCATCAGGCGCTAGGAAA	
e	TACTGGCCCTGCTCGATGTA	
f	TGCAATCAGGCGCTAGGAAA	
g	CTTCACGTGCCCATCAGCTC	
h	GCTAGATAGCGCTGCCTGTG	
i	GTACCGAAGTACGAGAGCCG	
j	GTAGGGGCAAGGACTGACAC	
k	TGTCGAGGGGTTGTACTCCA	
l	CTCGTCGGGGTGTCAGTTC	
p1	GGGTGTCGAGGGGTTGTACTC	Detection of integration of pIJintSt in *S. coelicolor*
p2	CCGTGCCAATCGGATCAGC	
b1	CTCACCGACAAGTCGATGC	
b2	CACAGGAGCGAGCGGGCAG	
piR	CCGTGCCAATCGGATCAGC	Detection of integration of pIJattR+ *in S. turgidiscabies* Car8
bsF	TGATCGTCGGCTCGATTCC	
bsR	GGATGAGCGGCTGCGTTTC	
piF	GGTTGGTAGGATCGTCTAGAACAGGA	
tomAF	TGGCTGACCACGGGCTTGC	Detection of *tomA* gene
tomAR	GCGAAGCCCCTCATGTTCG	
Nec1F	AATCGTGACTGTTTCATTC	Detection of *nec1* gene
Nec1R	AAATCCTTCCGCTGCGTTC	

Restriction and point mutation sites are indicated in bold.

Point mutations in the postulated catalytic region of *intSt* were constructed by PCR site-direct mutagenesis. Primers FintStYF with *intR* and RintStYF with *intF* (Table 2) were used to amplify the 5′ region and the 3′ region of *intSt*, respectively. We used overlapping PCR to join the amplification products and to generate the IntStY407F mutant. Applying the same strategy, we used a set of additional primers described in Table 2 to construct IntStR209K and IntStR375K. The resulting mutants were cloned into the NdeI-HindIII region of pIJamp001 to generate plasmids pIJintStY407F, pIJintStR375K, and pIJintStR209K (Table 1). Additional features of the plasmids are described in Table 1. Maps of the plasmids are provided in Figure S1.

### Mating Assays

*E. coli* ET12567 (Table 1) was transformed with plasmids by electroporation and was incubated on LB plates containing kanamycin, chloramphenicol and hygromycin B. Transformed *E. coli* isolates were grown in LB broth for 4 h at 37°C to an OD_600_ of 0.4–0.6. Ten ml of the suspension was then mixed with 0.5 ml of *Streptomyces* spores, concentrated to approximately 10^8^ spores per ml and incubated on MS agar supplemented with 10 mM MgCl_2_. After incubation for 24 h at 28°C, 1 ml of dH_2_O with nalidixic acid and hygromycin B was added to select for transconjugants.

### Amplification and Sequencing of Circular Structures and Recombination Sites

DNA samples were obtained from broth cultures after 24 h of incubation. DNA was isolated using the MasterPure™ Kit (Epicentre, Madison, WI, USA) according to the manufacturer's instructions. We used nested PCR to detect the recombination events within the *att* sites. Primers for each recombination event are described in Table 2. PCR reactions were conducted in volumes of 50 µl using 50 pmoles of each of the primers, 50 µg of genomic DNA, 1× of PCR Taq buffer New England Biolabs (Ipswich, MA, USA), 50 µM of dNTPs, 5% DMSO and two units of Taq DNA polymerase (New England Biolabs). Nested PCR was conducted as described above using 1 µl of the first PCR product as a template. Cycle conditions were: Initial denaturation, 94°C for 2 min followed by 35 cycles of denaturation at 94°C for 45 sec, annealing at 58°C for 45 sec; extension, 68°C for 90 sec, and final extension, 68°C for 5 min. Products were visualized by gel electrophoresis and gel purified for later sequencing.

### Detection of Integrated Plasmids in *S. coelicolor* and *S. turgidiscabies* Car8

DNA extraction was conducted as described above. PCR and Southern blot analysis were used to confirm the integration of the pIJintSt into the chromosome of *S. coelicolor* A3(2). Southern blot analysis was conducted by digestion of 4 µg of genome DNA with KpnI enzyme. Fragments were separated by electrophoresis in a 1% agarose gel. The DNA was depurinated with 0.1 M HCl, denatured with 0.5 NaOH and 1.5 M of NaCl, and neutralized with 1.5 M NaCl and 0.5 M Tris HCl. DNA samples were transferred to a nylon membrane. The membrane was probed with the PCR product obtained with primers intF and intR (Table 2). These primers amplify the total coding sequence (CDS) of *intSt* (predicted product 1,401 bp). The PCR product was labeled with dioxigenen-11-UTP Kit (Roche, USA). Hybridization was carried out at 58°C overnight and stripping conditions were carried out with 0.5× SSC solution at 62°C for 20 min.

Two pairs of primers, b1 with p2, and p1 with b2, were used to amplify by PCR the DNA structures of recombination formed by the integration process in *S. coelicolor* (Table 2). Primers b1 and p2 amplify the region that contains the 5′ end of the integrated pIJintSt plasmid. Primers p1 and b2 amplify the 3′ end of the integrated pIJintSt. Multiple integration events were detected using primers p1 and p2 (Table 2). Amplifications were conducted with a denaturation temperature of 94°C for 2 min, and 30 cycles of denaturation at 94°C for 45 sec, annealing at 58°C for 45 sec, and extension at 68°C for 60 sec, with a final extension of 68°C for 5 min. For detection of integration of pIJattR+ in *S. turgidiscabies* Car8, amplifications were carried out using the same conditions described above with primers bsF with piF, and piR with bsR (Table 2).

### Detection of *nec1, tomA* and *intSt*

Genomic DNA of selected strains (Table 3) was used as a template for the *nec1, tomA* and *intSt* genes; primers are described in Table 2. PCR conditions were: denaturation temperature of 94°C for 2 min, and 30 cycles of denaturation of 94°C for 45 sec, annealing at 60°C for 45 sec, extension at 68°C for 60 sec, and final extension at 68°C for 5 min. PCR products were visualized by electrophoresis in a 1% agarose gel.

**Table 3:** *Streptomyces* strains, geographical origins and PAI modules, as detected by PCR.

Strain	Geographical origins	*tomA/nec1* Module 105 Kb	*intSt* Module 569 Kb
***S. turgidiscabies***			
Car8	Japan	+/+	+
94-1	Japan	+/+	+
94-16	Japan	+/+	+
94-19	Japan	+/+	+
94-21	Japan	+/+	+
94-22	Japan	+/+	+
94-23	Japan	+/+	+
95-01	Japan	+/+	+
95-05	Japan	+/+	+
98-15	Japan	+/+	+
98-25	Japan	+/+	+
***S. scabies***			
87-22 (WT)	Wisconsin, USA	+/+	-
84-34 (ATCC49173)	Upstate NY, USA	+/+	-
84-70	Maine, USA	+/+	-
84-232	West Virginia, USA	+/+	-
85-08	Maine, USA	+/+	-
85-30	Ohio, USA	+/+	-
87-27	Wisconsin, USA	+/+	-
87-67	Wisconsin, USA	+/+	-
89-04	Alaska, USA	+/+	-
96-15	Germany	+/+	-
95-17	South Africa	+/+	-
96-06	Ontario, Canada	+/+	-
***S. acidiscabies***			
84-104	Maine, USA	+/+	-
84-45	Upstate NY, USA	+/+	-
84-110 (ATCC 49003)	Maine, USA	+/+	-
84-182	Maine, USA	+/+	-
85-06	West Virginia, USA	+/+	-
90-25	Maine, USA	+/+	-

PAISt, PAISs1 and PAISa1 share a module of 105 Kb. The modules were detected by PCR using primers for the *nec1* and *tomA* genes as markers. The *intSt* gene is encoded in the 569 Kb module of PAISt and is only present in *S. turgidiscabies* isolates but not in *S. scabies* and *S. acidiscabies*.

### Sequence Analysis

Comparative analysis of the genomic islands in *S. turgidiscabies* Car8 (PAISt), *S. scabies* 87–22 (PAISs1) and *S. acidiscabies* 84–104 (PAISa1) were conducted using the Artemis Comparison Tool (ACT) [15]. Sequences were retrieved from GenBank with the following accession numbers: PAISt (NZ_AEJB01000361.1); PAISs1 (NC_013929); PAISa1 (NZ_AHBF00000000). Comparative analysis of *intSt* (Genbank accession number WP_006379006) was conducted using six functionally characterized integrase sequences (Genbank accession numbers: YP_133692; NP_628777; CAA33029; NP_040609; NP_418256; ZP_01682564). These sequences were aligned to INTSt using the ClustalW2 alignment tool [16] in order to identify conserved domains. Structural modeling was carried out using EsyPred3D modeling server 1.0 [17]. Visualization of structures was completed using DeepView Swiss-PdbViewer v4.1 protein modeling software [18].

## Results

### PAISt Excises in Modules and Generates Circular Structures

**Figure 2:**
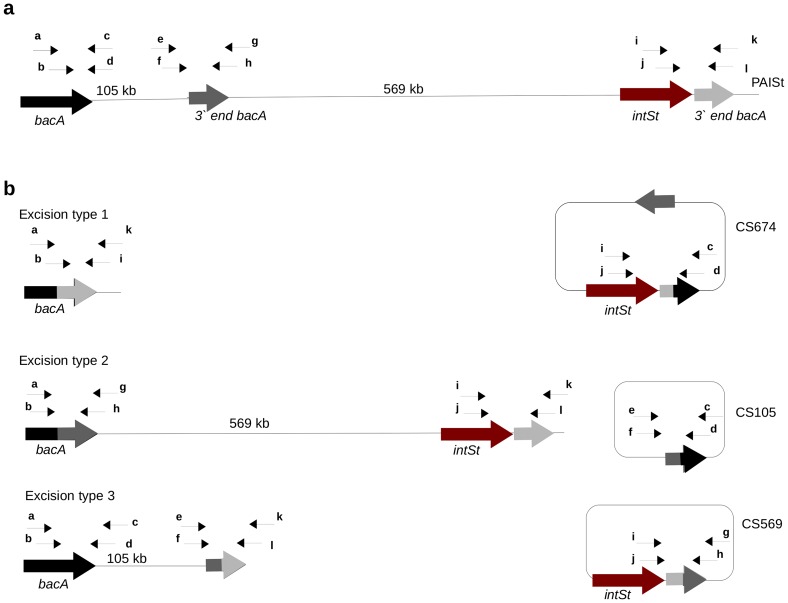
Schematic representation of the excision of circular structures from PAISt in *S. turgidiscabies* Car8. (**a**) PAISt and the location of the primers used for detection of specific recombination events within the *att* sites. Arrows indicate the locations of primers, and primer sequences are provided in Table 2. (**b**) Three types of excision resulting from recombination events at the *att* sites, which were detected using nested PCR. Excision type 1 produces CS674, excision type 2 produces CS105 and excision type 3 produces CS569.

**Figure 3:**
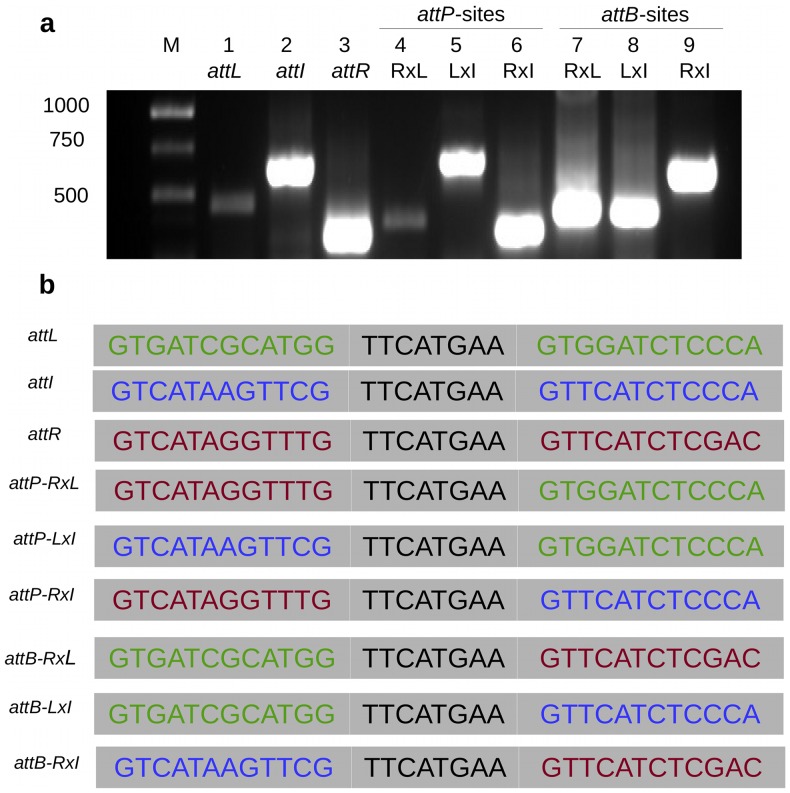
Amplification of the *attB* and *attP* sequences generated after the three types of excision from PAISt. Products were detected by nested PCR using primer pairs described in Table 2 and Figure 2. (**a**) PCR results resolved after electrophoresis in a 1% agarose gel. Lanes 1 to 3 show amplification of the *att* sites in the PAISt: *attL* (445 bp), *attI* (642 bp), and *attR* (341 bp). Lanes 4 to 6 show amplification of the *attP* sites in the circular structures (CS674, CS105 and CS569): recombination between *attR* and *attL* (*attP*-RxL, 380 bp), recombination between *attL* and *attI* (*att*P-LxI,663 bp), and recombination between *attR* and *attI* (*attP*-RxI, 359 bp). Lanes 7 to 9 shows amplification of the *attB* sites in the *S. turgidiscabies* chromosome: recombination between *attR* and *attL* (*attB*-RxL, 406 bp), recombination between *attL* and *attI* (*attB*-LxI, 424 bp), and recombination between *attR* and *attI* (*attB*-RxI, 624 bp). Lane M contains the 1 Kb ladder. (**b**) Sequences of the *att* sites with the palidromic sequences indicated in black. The flanking regions are presented in color to show recombination events within *att* sites (*attL* in green, *attI* in blue and *attR* in red).

Using a set of primers that target the junction regions of PAISt (Figure 2 and Table 2), we were able to detect recombination events between the flanking *att* sites (Figures 2 and 3). Nested-PCR results indicate the presence of at least three recombination events. The first event is the recombination between *attR* and *attL* (Figures 2 and 3a). This event produces the excision of the whole island as a circular structure of 674 Kb (CS674) with the recombination product *attP*-RxL and the formation of the recombination scar *attB*-RxL in the chromosome. These structures are detected as nested-PCR products of 380 bp for *attP*-RxL and 406 bp for *attB*-RxL (Figure 3a).

The second event is the recombination between *attL* and *attI* (Figures 2 and 3a). The recombination produces a circular structure of 105 Kb (CS105) with a recombination product *attP*-LxI and the scar *attB*-LxI in the chromosome. The structures are detected as products of 663 bp for *attP*-LxI and 424 bp for *attB*-LxI (Figures 2 and 3a). The third event is the recombination between *attR* and *attI* (Figures 2 and 3a). This recombination produces a circular structure of 569 Kb (CS569) with the recombination product *attP*-RxI and the scar *attB*-RxI in the chromosome. Nested PCR produces fragments of 359 bp (*attP*-RxI) and 624 bp (*attB*-RxI) (Figures 2 and 3a). Sequencing demonstrated that the 8 bp palindromic sequence occurred in all of the nested-PCR products, confirming that they represent a legitimate recombination event (Figure 3b).

### The Product of *intSt* Contains Motifs Conserved in Tyrosine-Recombinases

**Figure 4:**
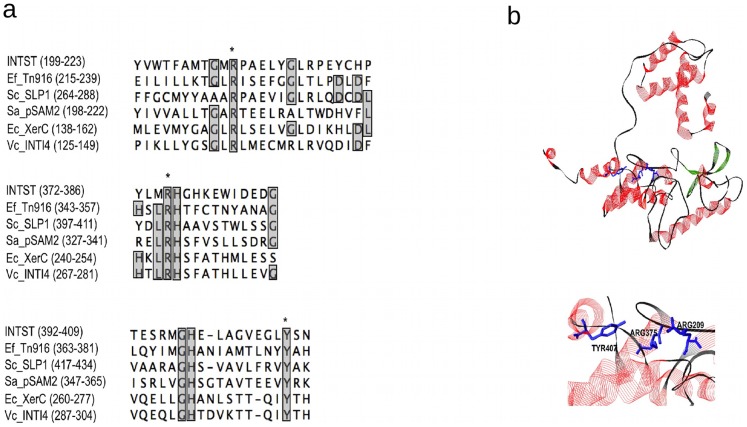
Sequence analysis and structure of *intSt*. (**a**) Amino acid alignment of INTSt with characterized integrases illustrating the three conserved regions. Conserved amino acids are indicated in grey. Asterisks indicate the amino acids R209, R375, and Y407. (**b**) Structural modeling of INTSt shows the residues RRY of the predicted catalytic site.

The 3′ end of PAISt contains a putative integrase *intSt* (Figure 1). The amino acid alignment of INTSt with experimentally characterized tyrosine recombinases demonstrates that discrete regions (boxes), typical of tyrosine recombinases, also occur in INTSt [19] (Figure 4a). The predicted conserved boxes in INTSt are located at amino acid positions 197 to 236 (Box A), 372 to 386 (Box B), and 392 to 409 (Box C). In addition, the amino acid catalytic triad of arginine, arginine, tyrosine (RRY), conserved in all tyrosine recombinases [19], is predicted to be located in the respective box regions at positions 209(R), 375(R) and 407(Y) (Figure 4a). Structural predictions of INTSt were carried out using *Vibrio cholerae* integrase INTI4 as a reference (GenBank accession ZP_01682564, protein structure code 3a2Vb). The 3D model of INTSt displays the RRY triad in a spatially associated cluster (Figure 4b).

### Characterization of Site-Specific Recombination Activity of INTSt in *Streptomyces* spp

**Figure 5:**
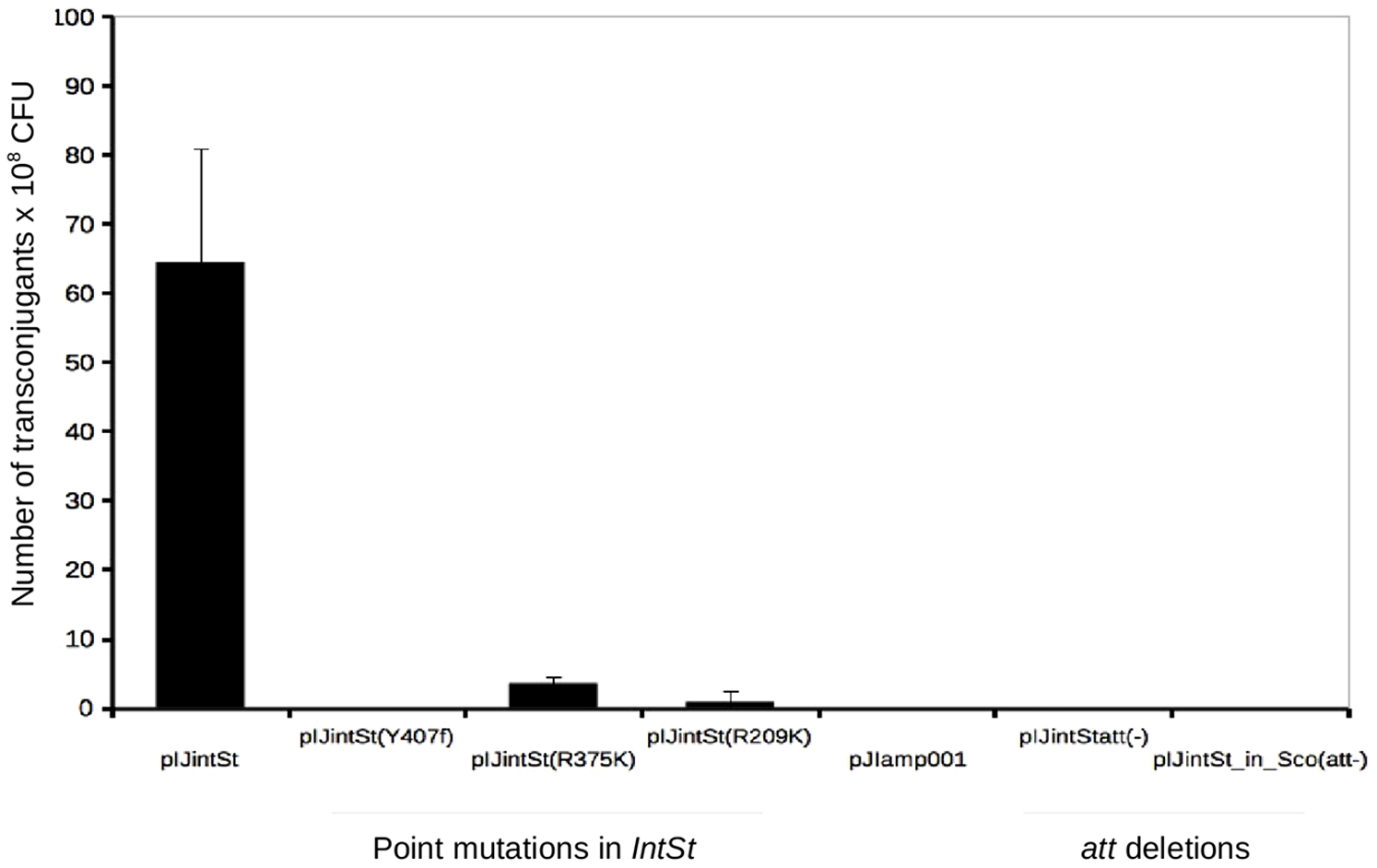
Integration activity of INTSt is affected by mutation in the catalytic core and deletions in the *att* sites. Frequencies of transconjugants obtained when pIJintSt was mobilized into *S. coelicolor* A3(2). Frequency of integration was calculated based on the number of hygromycin-resistant colonies (y-axis). Point mutations in the putative catalytic core of INTSt reduces integration events. Deletion of the *att* sites in the plasmid pIJintStatt(−) or in the chromosome of *S. coelicolor* Δ*bacA* (pIJintSt_in_Sco att-), abolishes integration events. Plasmid pIJamp001 was used to control for illegitimate recombination (recombination not mediated by INTSt). The data shown are the means of three independent experiments. Three colonies of *S. coelicolor* were selected and grown to obtain spore stocks. Spore stocks were tested with the integrative plasmids as described in material and methods. Means of the integration frequencies were plotted with error bars indicating the distribution of the data.

Previous experiments have shown that several *Streptomyces*, including *S. coelicolor*, are suitable hosts for the mobile PAISt [9]. We sought to confirm and characterize the recombination-integration activity of INTSt using the *S. coelicolor* chromosome as the target of integration. We mobilized pIJintSt into *S. coelicolor* by conjugation. pIJintSt cannot replicate in *Streptomyces* but represents a mini-PAISt with putative integration functions (See Materials and Methods). Integration events in *S. coelicolor* were obtained at an average frequency of 6.4 × 10^−7^ per recipient colony-forming unit (cfu) (Figure 5), when pIJintSt was mobilized from *E. coli* by conjugation.

We demonstrated that transconjugant frequency is highly affected either by point mutations within the putative catalytic core of *intSt*, or by mutations within the *att* sites. Three versions of pIJintSt vectors containing *intSt* with point mutations in codons for amino acid residues R209K, R375K, and Y407F, were not able to integrate into *S. coelicolor* (Figure 5); the mutated residues were predicted to form the catalytic region of the recombinase (Figure 4). Furthermore, the 8 bp palindrome is required for integration. The palindrome-less plasmid, pIJintSt*att*(−), was not able to integrate in the *S. coelicolor* chromosome and pIJintSt was not able to integrate in *S. coelicolor* Δ*bacA* (Figure 5).

**Figure 6:**
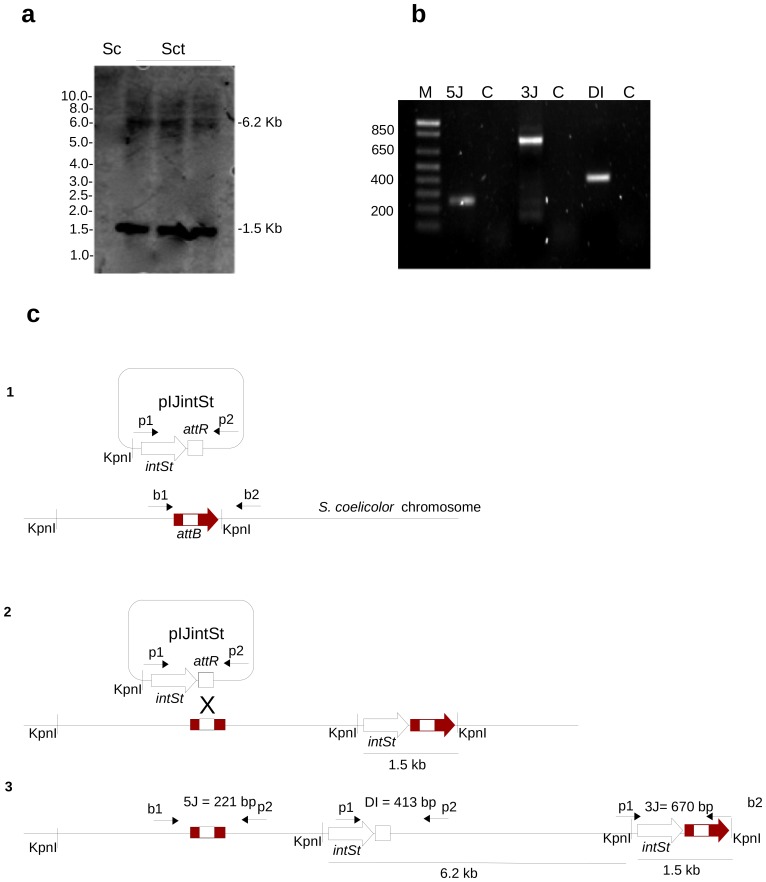
Detection of multimeric integration of pIJintSt in *S. coelicolor* A3(2). (**a**) Southern blot analysis shows three *S. coelicolor* A3(2) transconjugants (Sct). The blot was probed with a PCR product of *intSt*. Two hybridization signals are observed (1.5 Kb and 6.2 Kb). Sc indicates genomic DNA of *S. coelicolor* A3(2) wild type used as a control. Sizes corresponding to a 1 kb ladder are shown to the left of the southern blot. (**b**) PCR detection of the multimeric version of pIJintSt in *S. coelicolor* A3(2) transconjugants. Lane M contains the 100 bp DNA ladder. Lane 5J contains the junction formed at the 5′ end of the integration (detected with primers b1 and p2). Lane 3J contains the junction formed at the 3′ end of the integration (detected with primers b2 and p1). Lane DI contains the double integration event (detected with primers p1 and p2). Lane C is the DNA from the wild-type strain (negative control of the PCR reaction). (**c**) Detected events of pIJintSt integration in *S. coelicolor* A3(2) chromosome: figures 1 and 2 describe the integration at and duplication of the *att* site within *bacA*; figure 3 shows a second integration and the formation of a multimeric version of pIJintSt. Small black arrows indicate primers used to detect each event (Table 2). The predicted PCR product sizes are indicated. The KpnI restriction sites are indicated and correspond to the Southern blot results provided in panel **a**.

As expected, integration of pIJintSt occurs at the 3′ end of *bacA*. However, Southern blot analysis of transconjugants revealed the presence of tandem repeats of pIJintSt at the integration site in *S. coelicolor*. Two bands of 6.2 Kb and 1.5 Kb were detected when they were digested with KpnI and probed with *intSt* (Figure 6a). The multiple recombination events were also confirmed with PCR (Figures 6b and 6c).

### PAISt Integrates Circular Plasmids by Site-Specific Recombination at the 3′ end of *bacA*

**Figure 7:**
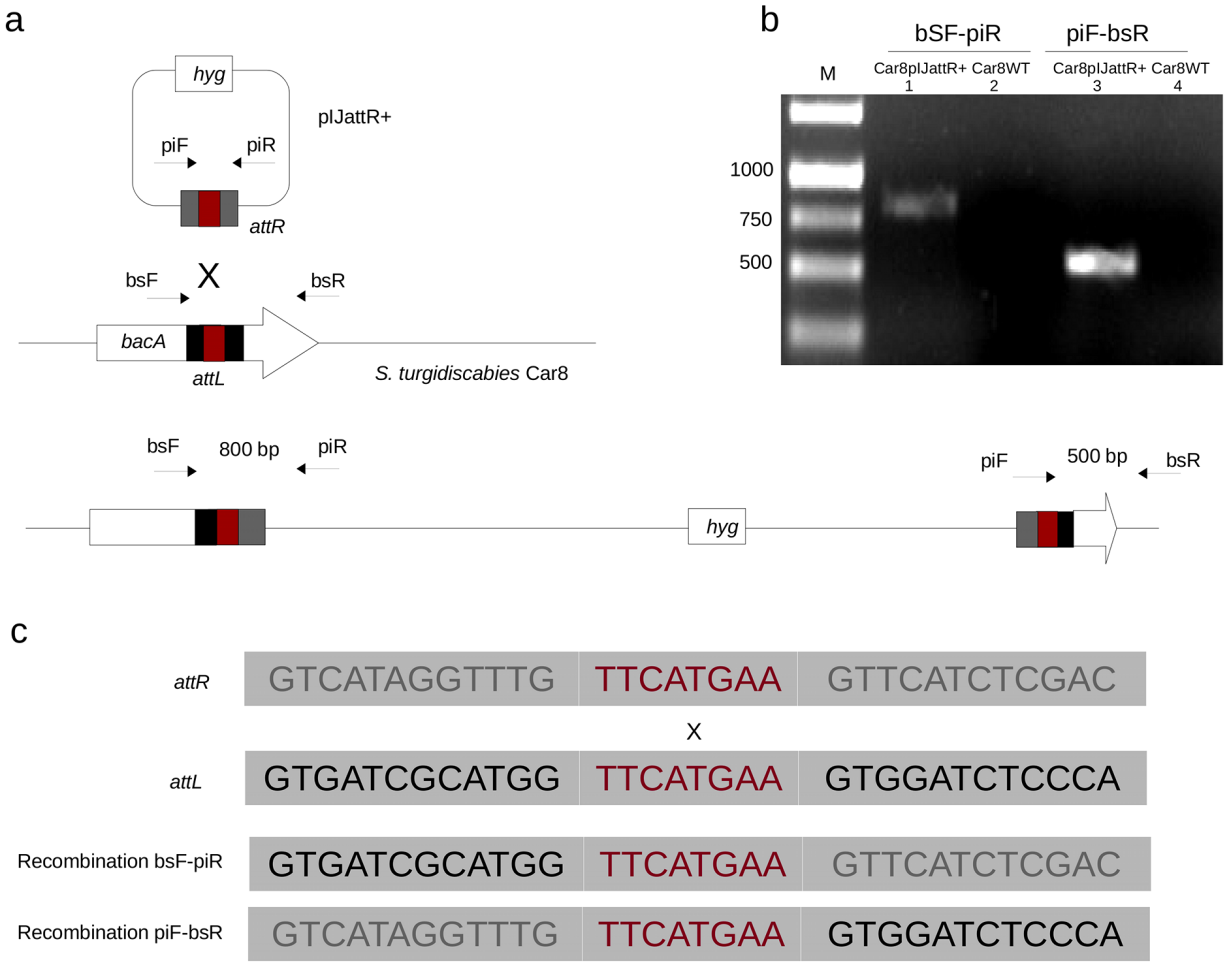
Integration of plasmids containing *att* sites into the chromosome of *S. turgidiscabies* Car8. (**a**) pIJattR+ integrated at *bacA* in the *S. turgidiscabies* Car8 chromosome. Primers used to detect integration events and their products are shown (**b**) Amplification of the integration junctions. Lane M contains the 1 kb DNA ladder. Lanes 1 and 2 contain the products of the left junction (amplified with primers bSF-piR). Lanes 3 and 4 contain the products of the right junction (amplified with primers piF-bsR). Car8pIJattR+ is the transconjugant and Car8WT is the *S. turgidicabies* Car8 wild type used as a control. (**c**) Sequences of PCR products. The 8 bp palindrome and the flanking sequences of each PCR product are aligned.

Integration of circular plasmids containing *att* sites was observed in *S. turgidiscabies* Car8. These events were detected when plasmid pIJamp001, containing the *attR* site of PAISt (pIJattR+), was conjugated from *E. coli* to *S. turgidiscabies* Car8. Plasmid pIJattR+ contains the hygromycin B resistance cassette and can replicate in *E. coli* but not in *S. turgidiscabies* Car8. Detection of hygromycin B resistant colonies indicated integration of pIJattR+. PCR amplification and sequencing confirmed that pIJattR+ integrates at the 3′ end of *bacA*, suggesting recombination with the *attL* of PAISt (Figure 7). Two PCR products of 800 bp and 500 bp were detected using primers that flank the junctions of the integration of pIJattR+ in the *attL* site of *S. turgidiscabies* Car8 (Figure 7). Transconjugants (Car8pIJattR+) were observed at an average frequency of 3×10^−8^ per recipient cfu. Integration in *S. turgidiscabies* Car8 was not observed when a pIJamp001 (*attR*-less version) was used in conjugation assays. These results suggest that integration depends on the presence of *att* sites.

### Comparative Genome Analysis Suggests the Presence of Remnants of *intSt*

**Figure 8:**
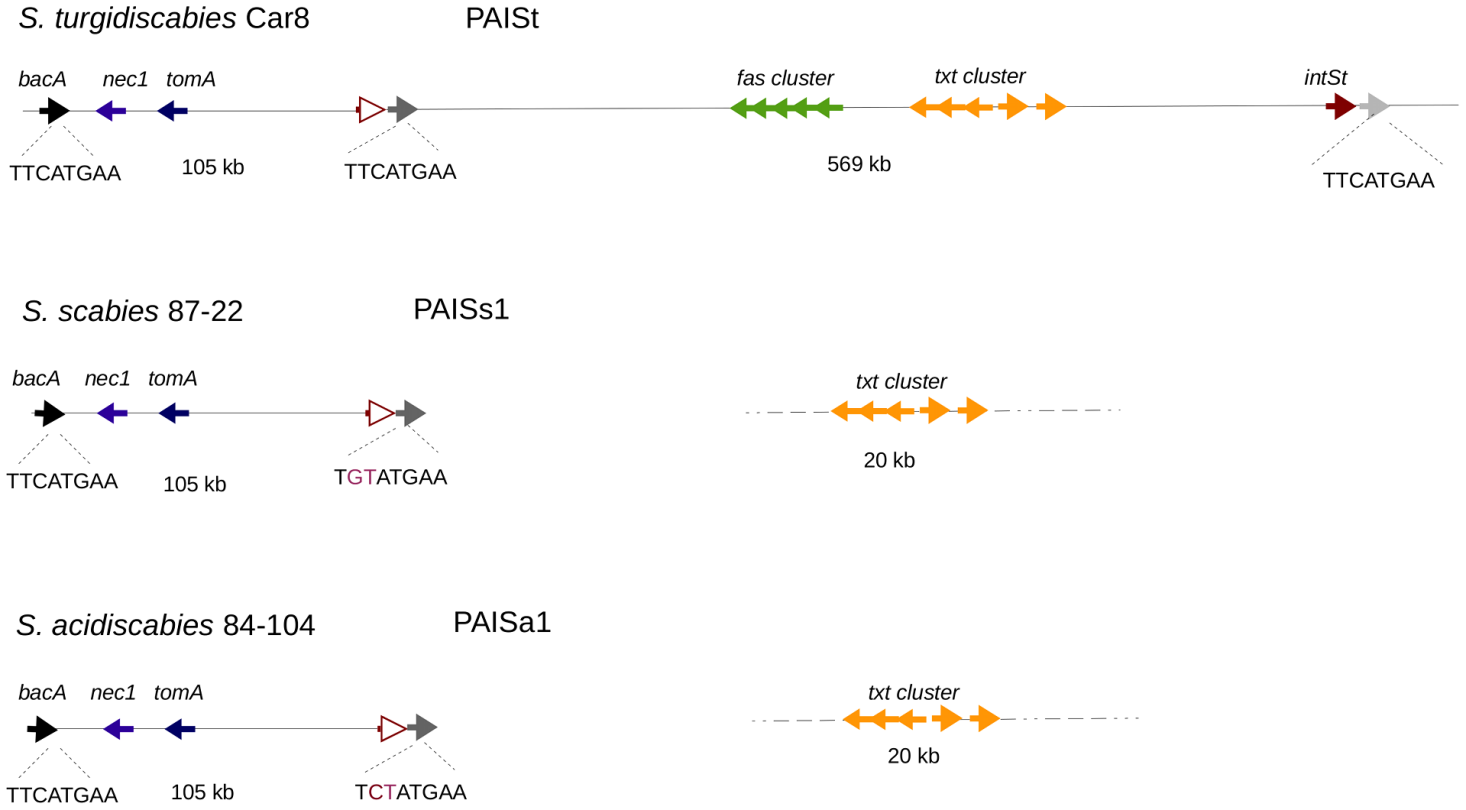
Relationships of PAISt with PAISs1 in *S. scabies* 87–22 and PAISa1 in *S. acidiscabies* 84–104. Islands PAISs1 in *S. scabies* and PAISa1 in *S. acidiscabies*, are integrated at the *bacA* 3′ end and contain a remnant of the *intSt* (red line arrow) delimited by a degenerate version of the 8 bp palindrome, (mutated residues are in red). Both islands are identical to the 105 Kb module of PAISt. The thaxtomin biosynthesis cluster (*txt*) is conserved in *S. scabies* and *S. acidiscabies* but is not linked to the 105 Kb island.

Comparative genome analysis of *S. turgidiscabies* Car8 with other pathogenic *Streptomyces* species revealed that the 105 Kb module of PAISt was identical to islands PAISs1 in *S. scabies* 87–22 and PAISa1 in *S. acidiscabies* 84–104 (Figure 8). Both islands were inserted at *bacA* and were delimited by a 138 bp copy of the *bacA* 3′ end (Figure 8). In contrast, the 569 Kb region of PAISt was missing in *S. scabies* and *S. acidiscabies*, with the exception of the 20 Kb region coding for the thaxtomin biosynthesis cluster, which was not linked to the 105 Kb module.

**Figure 9:**
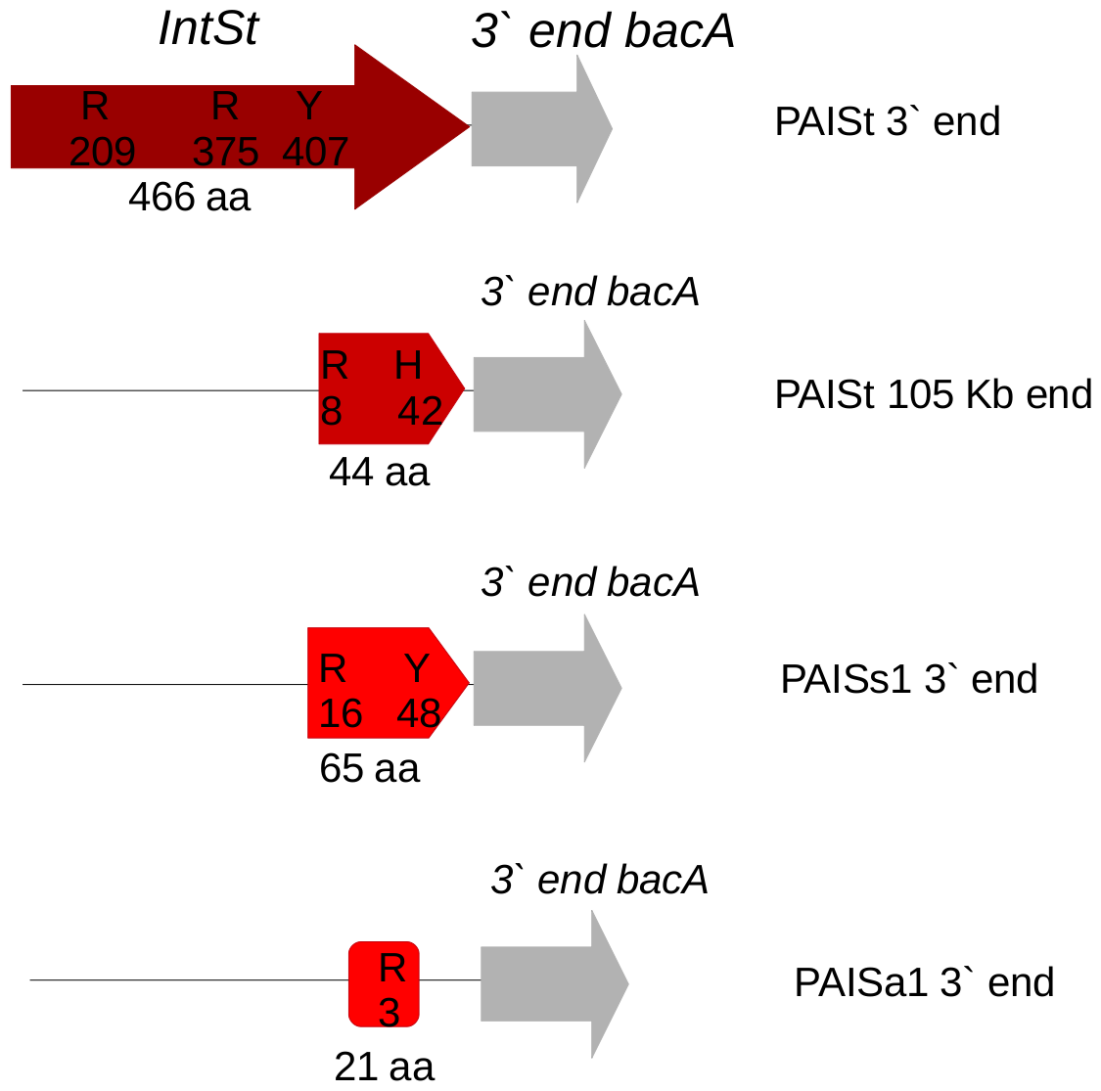
Remnants of *intSt* exist in PAISt, PAISs1 and PAISa1. Alignment of INTSt with three frame translations of the 3′ end of the first 105 Kb module of PAISt and with the 3′ ends of PAISs1 and PAISa1 reveal INTSt remnants. Remnants are shown as truncated red arrows. Conserved amino acids residues and their positions in the alignment are indicated.

Alignment of INTSt with the six frames of the 3′ ends of PAISa1, PAISs1 and the middle *att* site of PAISt (*attI*) showed that small remnants of *intSt* are found in these regions (Figure 9). All the remnants aligned with C-terminal region of INTSt but lacked at least one conserved catalytic residue observed in *intSt* (Figure 9). In the case of the middle *attI* region of PAISt, we found a 44 aa remnant of INTSt. The remnant contained the arginine (R8) residue corresponding to the R375 in INTSt and a mutation of the tyrosine (Y407) in INTSt to histidine (H42). In *S. scabies* 87–22, the 3′ end of PAISs1 displayed a 65 aa remnant of INTSt. This remnant contained the catalytic residues R375 and Y407 conserved at positions R16 and Y48 (Figure 9). In *S. acidiscabies* 84–104, the 3′ end of PAISa1 displayed a remnant of 21 aa. This remnant contained the conserved arginine residue (R375) at position 3 of the remnant (Figure 9).

Islands PAISs1 in *S. scabies* 87–22 and PAISa1 in *S. acidiscabies* 84–104 had imperfect palindromic sequences at their 3′ ends. In comparison to the conserved palindromic 8 bp observed in PAISt, PAISs1 contained an imperfect palindrome with two point mutations (TGTATGAA). In the case of PAISa1, the 3′ end contained one point mutation (TCTATGAA) (Figure 9). We did not detect recombination events between the flanking regions of the islands in either *S. scabies* 87–22 or *S. acidiscabies* 84–104 (data not shown). These results contrast with *S. turgidiscabies* Car8, which contains a complete coding integrase and intact palindromic regions. Furthermore, integration of pIJattR+ was not detected in *S. scabies* 87–22 or *S. acidiscabies* 84–104 (data not shown).

It is important to note that all other strains of *S. turgidiscabies* examined contained the three landmarks of PAISt: *intSt*, *nec1*, and *tomA*, (Table 3). Similarly, other isolates of *S. scabies* and *S. acidiscabies* contained *nec1* and *tomA* but lacked *intSt*. These data suggest the conservation of the 105 Kb module of the island in the three species (Table 3).

## Discussion

Our results confirm the presence of extra-chromosomal circular structures in *S. turgidiscabies* Car8. We were able to demonstrate the presence of discrete deletions within the PAISt island due to excision. These circular structures are the product of recombination between the PAISt *att* sites. Mating of *S. turgidiscabies* Car8 with other *Streptomyces* is not a requirement for circular structure formation, as the structures were detected when *S. turgidiscabies* was grown in broth cultures.

Modular excision of genome islands in pIant pathogenic *Streptomyces* species could be a significant source of genome plasticity. In Figure 3a, amplification products in lanes 1 and 4 appear to be less abundant than in lanes 2, 3 and 5 to 9. We suspect that this indicates that there is another layer of complexity that influences the relative copy number of the circular structures and the chromosomally integrated versions of these circular structures. For example, excision and recombination events might occur in a stochastic manner. Re-integration and stability of individual circular structures might differ. Finally levels of excision and integration might depend on the presence of additional factors. Excision of several ICEs is modulated through interaction with an excisionase protein, Xis, with the integrase [20], [21].

In PAISt a small region of 557 bp, coding for a putative Xis (Genbank accession number WP_006378952) is located just upstream of the *intSt*. It might be that interaction of Xis with INTSt plays a role in the excision of PAISt. Additional research is needed to unravel the dynamics of excision and integration of circular structures.

Modular excision of ICEs has been reported in other bacteria. Frankia sp. possesses the ICE Fean6303, which contains three direct repeated sequences, two flanking and one internal direct repeat sequences (*attL, attL2* and *attR*). Fean6303 excises by recombination between *attR* and either *attL* or *attL2* [22]. Similar observations have been reported for the element ICE*St1* in *Streptococcus thermophilus* [23] and the pathogenicity islands, ICEE*c*1 in *Escherichia coli* [24] and ROD21 in *Salmonella enterica* serovar enteritidis [25]. For the specific case of *S. enterica*, excision of ROD21 results in the loss of the island from the chromosome and reduced virulence [25]. It is possible to extrapolate this scenario to *S. turgidiscabies*, and suggest that there may be more than one version of PAISt in other strains, and as a result these strains might have different virulence characteristics. Further analysis of other PAISt homologs in plant pathogenic *Streptomyces* isolates could reveal a link between the variability of the island and associated levels of virulence.

We demonstrate that *intSt* can integrate plasmids in tandem arrays. The presence of these structures, flanked by *att* sites, suggests several scenarios. First, the tandem arrays may form after the transfer of pIJintSt concatemers from *E. coli* into *S. coelicolor*. An alternative possibility is the transfer of single pIJintSt from multiple plasmid donors into *S. coelicolor* and subsequent integration. Cell development of *Streptomyces* may provide yet another explanation. Multiple single copies of pIJintSt may successively mobilize through the undifferentiated hyphae in the mycelium and integrate in the chromosome. The process of multiple recombination events is not unique to PAISt. The observation of tandem arrays has been described in the ICEs SXT and R391 which occur in *Vibrio cholerae* [26].

Other important characteristic of PAISt is the ability to capture plasmids containing the *att* sites. The integration of plasmids at the *attL* site of PAISt resembles a site-specific recombination mechanism in integrons [27], [28]. These genomic elements are able to “capture” and integrate gene cassettes into a host chromosome, using an integrase and a cognate recombination site [27]. Properties of capture, modular excision, and mobilization define PAISt as a “mobile gene collector” and likely plays an important role in shaping the genome of plant pathogenic *Streptomyces*. In our experiments, we cloned the *attR* site of PAISt in pIJamp001 and used this *att* site as an integration substrate. As observed in *S. coelicolor*, we detected integration only between the pIJattR+ and the *attL* site (3′ end of the *bacA*).

The activity of INTSt and the recognition of the recombination sites are also worthy of consideration. Tyrosine recombinases follow a specific chemistry in which the tyrosine residue carries out a nucleophilic attack of the target sequences to produce the cleavage. Sequence alignment, modeling of INTSt point mutation analysis allowed us to identify the putative nucleophilic tyrosine and the arginine residues that comprise the catalytic region of INTSt. Interestingly, INTSt is not identified as an integrase in the Pfam [29] or COG [30] databases. Furthermore, several homologs of INTSt are annotated as hypothetical proteins, not integrases, in the Genbank database. This suggests that INTSt represents a novel group of integrases that should be added to this protein family.

Although the minimal requirement for functional *att* sites has not been defined in this study, our results suggest that nucleophilic attack and cleavage occurs within the 8 bp palindromic sequence (Figure 3B). However, we believe that additional sequence requirements must be important for the recognition of the *att* sites. For instance, the *attR* site in the plasmid was able to integrate into the *attL* site in the chromosome, but we did not observe integration into the chromosomal copies of *attI* or *attR*. Furthermore, the *S. turgidiscabies* Car8 chromosome contains 178 copies of the 8 bp palindromic sequence, but integration occurred only at *attL* within the 3′ end of *bacA*. In tyrosine recombinases the *att* sites are typically between 30 to 200 bp and consist of two partial inverted-repeats that flank a central crossover sequence at which the recombination takes place [31]. Despite the fact we observed that the central crossover sequence for INTSt is the 8 bp palindrome, future research should address the accurate definition of the *att* sites and the exact chemistry of the INTSt.

The presence of remnants of *intSt* in sedentary islands of *S. scabies* and *S. acidiscabies* suggests a role for *intSt* in the excision and integration of PAISt. Furthermore, comparative sequence analysis of PAISt suggests several possible scenarios related to the evolution of PAISt and its homologs in other plant pathogenic *Streptomyces*. It is possible that in the case of *S. scabies* 87–22 and *S. acidiscabies* 84–104, the 105 Kb module of PAISt might have encoded an active *IntSt* and recombined in *bacA*. Once integrated in the host chromosome, the island underwent fixation. Gene erosion in the integrase and the *attR* (the integrative/excision module) may have caused the current sedentary status of the element in *S. scabies* 87–22 and *S. acidiscabies* 84–104 (Figure 8). In addition the presence of the thaxtomin cluster in *S. scabies* and *S. acidiscabies* indicates that this region might have self-mobilization and integration properties. The observations in *S. scabies* and *S. acidiscabies* can be understood as multiple and independent processes of lateral gene transfer in these two *Streptomyces*. By contrast, *S. turgidiscabies* Car8 represents an instance in which the element is still mobile and able to cause chromosomal rearrangements. PAISt contains a pseudo-*intSt* followed by the *attI* site (Figures 8 and 9), suggesting that more than one recombination event might have led to the current status of this element in *S. turgidiscabies* Car8.

Two recombination models might explain the mechanism of expansion of PAISt. Since the 3′ end of the *bacA* gene contains the 8 bp palindrome (*attI*), a second site-specific recombination at the *att* sites could have caused the expansion of PAISt. Since our results indicate that *intSt* is able to facilitate the creation of tandem arrays of plasmids, and PAISt behaves as an active integron, it is likely that site-specific recombination events may have affected the evolution of the island in *S. turgidiscabies* Car8. However, it is possible that the expansion may have happened via homologous recombination of a 105 Kb module with a second mobile island that shared sufficient sequence similarity.

We believe that PAISt is a dynamic structure able to excise, mobilize, and capture DNA. These features impact the evolution of *S. turgidiscabies* genome. The presence of a modular island may provide *S. turgidiscabies* an evolutionary advantage. Production of some virulence-associated metabolites may be metabolically costly but necessary in certain conditions when interacting with plants. We observed that PAISt “shapes” the chromosome of its host and produces gene content variability. The iterative excision and integration of this genomic element may facilitate the adaption of the host strain's metabolism to saprophytic and pathogenic life styles. The absence of the integrase *intSt* in *S. scabies* and *S. acidiscabies* strains isolated from different locations suggests that integrase and *att* site erosion are generalized processes in these species. Further characterization of pathogenic streptomycete genomes is likely to identify new versions of PAISt, allowing us to define the core genes of the element and to investigate the expansion hypothesis.

## Supporting Information

Figure S1 **Map of the plasmids used in this study.** All the plasmids are derivatives of pIJ10257 and are described in Table 1. *oriT* is the origin of transfer; *hyg* is the hygromycin resistance gene; *amp* is the ampicillin resistance gene, *ermE*p* is the constitutive strong promoter described in materials and methods.
(TIF)
