# Correction: Characterization of the Integration and Modular Excision of the Integrative Conjugative Element PAISt in *Streptomyces turgidiscabies* Car8

**DOI:** 10.1371/journal.pone.0121152

**Published:** 2015-04-01

**Authors:** The PLOS ONE Staff

In Fig. 1, the names of the genes nec1 and tomA are swapped. Please see the corrected Fig. 1 here.

**Fig 1:**
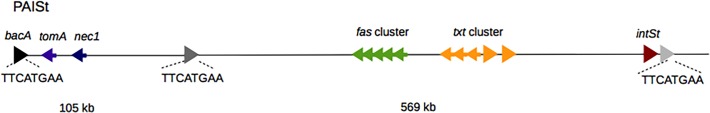
Schematic representation of the PAISt in *S*. *turgidiscabies* Car8. Copies of the 3′ end of the bacitracin resistance gene (*bacA*) delimit the element in two modules of 105 Kb and 569 Kb. The virulence genes *nec1* and *tomA* are located in the first module and the fasciation (*fas*) and thaxtomin (*txt*) biosynthetic clusters are located in the second module. The putative integrase (*intSt*) is located at the 3′ end of the island. The 8 bp palidromic repeats are shown within the *bacA* gene and its truncated copies.

In Fig. 8, the names of the genes nec1 and tomA are swapped. Please see the corrected Fig. 8 here.

**Fig 8:**
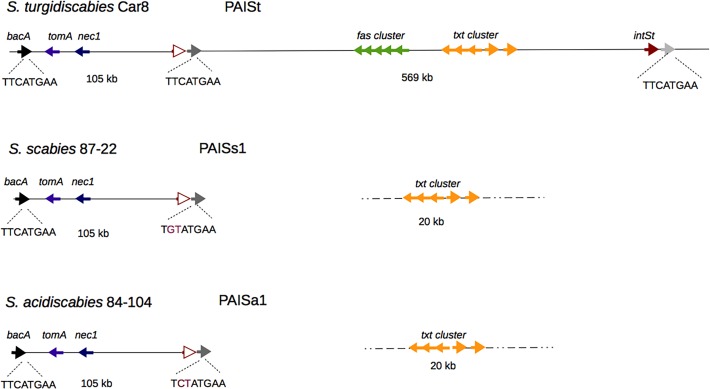
Relationships of PAISt with PAISs1 in *S*. *scabies* 87–22 and PAISa1 in *S*. *acidiscabies* 84–104. Islands PAISs1 in *S*. *scabies* and PAISa1 in *S*. *acidiscabies*, are integrated at the *bacA* 3′ end and contain a remnant of the *intSt* (red line arrow) delimited by a degenerate version of the 8 bp palindrome, (mutated residues are in red). Both islands are identical to the 105 Kb module of PAISt. The thaxtomin biosynthesis cluster (*txt*) is conserved in*S*. *scabies* and *S*. *acidiscabies* but is not linked to the 105 Kb island.
