# Supplementary figures and images for: Characterization of the Integration and Modular Excision of the Integrative Conjugative Element PAISt in Streptomyces turgidiscabies Car8
Source: PLoS One. 2014 Jun 13;9(6):e99345. doi: 10.1371/journal.pone.0099345 (PMC4057263; doi:10.1371/journal.pone.0099345)

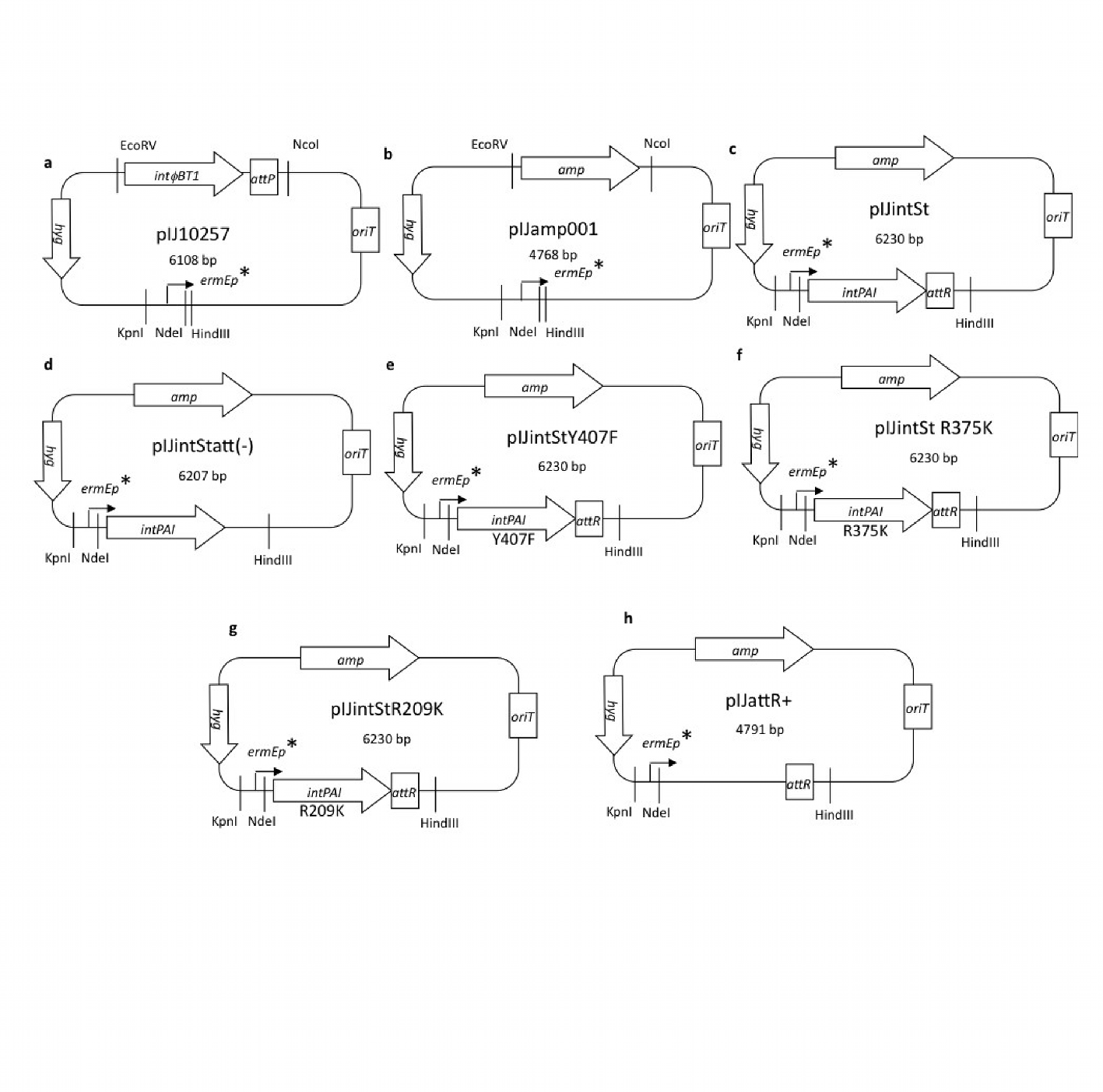

Supplement: Figure S1 — Map of the plasmids used in this study. All the plasmids are derivatives of pIJ10257 and are described in Table 1. oriT is the origin of transfer; hyg is the hygromycin resistance gene; amp is the ampicillin resistance gene, ermEp* is the constitutive strong promoter described in materials and methods. (TIF) [file pone.0099345.s001.tif]
